# Detoxification of reactive oxygen species by the hyaluronic acid capsule of group A *Streptococcus*

**DOI:** 10.1128/iai.00258-23

**Published:** 2023-10-24

**Authors:** Shyra Wilde, Ananya Dash, Anders Johnson, Kialani Mackey, Cheryl Y. M. Okumura, Christopher N. LaRock

**Affiliations:** 1 Microbiology and Molecular Genetics Program, Graduate Division of Biological and Biomedical Sciences, Laney Graduate School, Emory University, Atlanta, Georgia, USA; 2 Immunology and Molecular Pathogenesis Program, Graduate Division of Biological and Biomedical Sciences, Laney Graduate School, Emory University, Atlanta, Georgia, USA; 3 Department of Biology, Occidental College, Los Angeles, California, USA; 4 Department of Microbiology and Immunology, Emory University School of Medicine, Atlanta, Georgia, USA; 5 Division of Infectious Diseases, Department of Medicine, Emory University School of Medicine, Atlanta, Georgia, USA; 6 Antibiotic Resistance Center, Emory University School of Medicine, Atlanta, Georgia, USA; University of Illinois, Chicago, Illinois, USA

**Keywords:** *Streptococcus pyogenes*, group A *Streptococcus*, capsule, interleukins, oxygen radicals

## Abstract

The pro-inflammatory cytokine IL-6 regulates antimicrobial responses that are broadly crucial in the defense against infection. Our prior work shows that IL-6 promotes the killing of the M4 serotype group A *Streptococcus* (GAS) but does not impact the globally disseminated M1T1 serotype associated with invasive infections. Using *in vitro* and *in vivo* infection models, we show that IL-6 induces phagocyte reactive oxygen species (ROS) that are responsible for the differential susceptibility of M4 and M1T1 GAS to IL-6-mediated defenses. Clinical isolates naturally deficient in capsule, or M1T1 strains deficient in capsule production, are sensitive to this ROS killing. The GAS capsule is made of hyaluronic acid, an antioxidant that detoxifies ROS and can protect acapsular M4 GAS when added exogenously. During *in vitro* interactions with macrophages and neutrophils, acapsular GAS can also be rescued with the antioxidant N-acetylcysteine, suggesting this is a major virulence contribution of the capsule. In an intradermal infection model with *gp91^phox^* ^-/-^ (chronic granulomatous disease [CGD]) mice, phagocyte ROS production had a modest effect on bacterial proliferation and the cytokine response but significantly limited the size of the bacterial lesion in the skin. These data suggest that the capsule broadly provides enhanced resistance to phagocyte ROS but is not essential for invasive infection. Since capsule-deficient strains are observed across several GAS serotypes and are competent for transmission and both mild and invasive infections, additional host or microbe factors may contribute to ROS detoxification during GAS infections.

## INTRODUCTION

*Streptococcus pyogenes* (group A *Streptococcus* [GAS]) is a significant cause of pharyngitis (strep throat) and skin infections that are a major global disease burden (1). Furthermore, mild GAS infections can trigger immune complications such as rheumatic heart disease or develop into more severe diseases such as scarlet fever, sepsis, puerperal fever, toxic shock syndrome, or necrotizing fasciitis, collectively responsible for an estimated 500,000 annual deaths (1). There is currently no vaccine, and while GAS remains sensitive to several antibiotics, treatment failure remains a challenge (2).

Despite the potential for severe disease, most interactions of GAS with its host occur with little to no disease and resolve without further progression due to the effectiveness of the human immune system. Timely initiation of inflammation is essential in effectively coordinating the immune response during infection and its resolution (3). For example, anti-inflammatory medications that therapeutically interfere with signaling by the proinflammatory cytokine IL-1β are associated with an increased incidence of severe GAS infection in humans (4). IL-6, another cytokine of the acute inflammatory response, is also broadly crucial in restricting infections and is strongly induced during GAS infection (4 – 6). Within phagocytes, IL-6 signaling activates multiple transcriptional regulators, including STAT3 and NF-kB, that are important in immunity (7). Like with IL-1, excessive IL-6 can have pathological effects that result in autoimmune disorders such as rheumatoid arthritis and Castleman’s disease (8). Humans on IL-6-inhibiting biologic drugs have elevated reporting of invasive GAS infections, and mice treated with IL-6-inhibitors or that have *il6* gene knockout (IL6^-/-^) succumb to systemic GAS infections faster than wild-type mice (9).

We previously observed that the essentiality of IL-6 is variable during infections by different clones of GAS, unlike what was observed with IL-1, which has an invariant role in immunity between GAS strains (4, 9). The globally disseminated hypervirulent M1T1 serotype was fully resistant to IL-6-regulated immunity (9). In contrast, an M4 clone isolated from a pediatric patient, M4C20, who had received immunotherapy against IL-1 and IL-6 was killed in an IL-6-dependent manner (9). Therefore, greater resistance to IL-6-mediated immune responses can provide a selective advantage during invasive infections and is encoded by M1T1 GAS but not M4. A notable characteristic of M4 GAS, including the strain M4C20, is an absence of the *hasABC* genetic locus that is conserved in nearly all other GAS serotypes (10). These genes are essential for synthesizing hyaluronic acid (HA), the high molecular weight polymer of glucuronic acid and N-acetyl-glucosamine repeats that GAS uses to build its capsule (11). In GAS strains that encode it, the capsule promotes cell binding, antagonizes phagocytosis, and promotes resistance to immune antimicrobials for an overall contribution to bacterial survival in several *in vivo* infection models (12 – 16). Some polysaccharide capsules, such as those of *Cryptococcus neoformans* (17), *Salmonella enterica* (18), and *Streptococcus pneumoniae* (19) increase resistance against H_2_O_2_ up to millimolar levels. However, this is not a universally conserved property of capsules, as those of other species such as *Acinetobacter baumannii* (20), *Streptococcus equi* (21), and *Streptococcus iniae* (22) have lesser effect.

This work shows that the capsule protects against reactive oxygen species (ROS) induced by IL-6. Mechanistically, the hyaluronic acid capsule produced by some GAS strains protects against ROS by acting as a direct antioxidant. *In vitro*, strains of GAS that do not produce hyaluronic acid are more susceptible to hydrogen peroxide, but growth is rescued when exogenous hyaluronic acid is added. However, we find that the capsule is not essential for protecting against killing by phagocyte ROS in a mouse skin infection model. This work enhances our understanding of the immune processes that drive invasive skin infections caused by GAS.

## MATERIALS AND METHODS

### Bacterial culturing and strains

GAS strains 5448, 5448∆*hasA*, and M4C20 have been characterized previously (9, 13, 23). All other GAS clinical isolates were obtained through the Georgia Emerging Infections Program. Bacteria were routinely grown overnight in Todd Hewitt broth (BD) at 37°C with 5% CO_2_. For SpeB expression assays, supernatants were taken from freshly grown overnight cultures. For *in vitro* experiments, cultures were washed in phosphate-buffered saline (PBS) and resuspended in PBS + 20% glycerol and then stored in single-use aliquots at –80°C for later use. Bacterial aliquots were diluted to the appropriate multiplicity of infection (MOI) at the time of the experiment.

### Hydrogen peroxide susceptibility assays

Susceptibility to hydrogen peroxide was assessed using both solid and liquid media. For the solid media method, 0.1 mL of GAS in Todd Hewitt plus yeast (THY) broth was spread onto a THY agar plate and left to dry completely. When plates were sufficiently dried, 1 µL of 30% hydrogen peroxide was spotted onto the plate in triplicate, and the plates were incubated overnight. The following day, susceptibility was measured as the zone of inhibition surrounding the peroxide. A second iteration of this assay was performed using sterile paper filter discs soaked in either distilled water (control), 30% hydrogen peroxide, or a 1:1 solution of 30% hydrogen peroxide and 1 mg/mL of HA (Sigma). Zones of inhibition for each condition were quantified after imaging.

### Minimum inhibitory concentration

Minimum inhibitory concentration (MIC) assays using liquid media were carried out in 96-well plates using a method adapted from Ayala and Shafer (24). Briefly, dilutions of hydrogen peroxide were made in RPMI 1640 media (no fetal bovine serum [FBS], no antibiotics, no phenol red; Gibco), 5 × 10^5^ CFU was added, then the mixture was incubated at 37°C, 5% CO_2_ for 18 hours. The following day, alamarBlue (Bio-Rad), a colorimetric reagent for measuring cell respiration, was added at a 1:10 dilution and incubated at 37°C for 2–4 hours to allow for color development. The MIC was determined to be the lowest concentration that inhibited the growth of GAS (media remained blue after the addition of alamarBlue). Each strain was tested three times to ensure reproducibility.

### Capsule protection assays

Assays were performed as described in Brissac et al. with slight modifications (25). In addition, 5 × 10^7^ CFU of GAS were washed in 1-mL PBS and resuspended in RPMI containing no FBS or antibiotics with 0.05% Tween 20 (Sigma). Streptococcal hyaluronic acid (Sigma) was added to bacterial suspensions to a final concentration of 0.1, 1, or 2 mg/mL. After mixing by vortexing, H_2_O_2_ was added to a final concentration of 10 mM in each tube. The tubes were incubated for 30 minutes at 37°C in parallel with an H_2_O_2_-only (0-mg/mL HA) and an untreated control group, and CFU was enumerated by dilution plating.

### Cell culture

THP-1 monocytes (human; ATCC, TIB-202) were cultured at 37°C in 5% CO_2_ using RPMI with phenol red supplemented with 10% FBS and penicillin/streptomycin. Monocytes were terminally differentiated into macrophages using phorbol 12-myristate 13-acetate (PMA; 200 nM) for 48 hours before experiments as done previously (9).

### Gentamicin protection assays

THP-1 monocytes were seeded as 0.1 mL at 2 × 10^5^ cells/mL in a 96-well plate and terminally differentiated with 200-nM PMA for 48 hours. On the day of infection, cell culture media was replaced with RPMI (+FBS/−antibiotics; Gibco) supplemented with 10% pooled human serum (Lot # C16037, Atlanta Biologicals). At this time, wells were treated with N-acetylcysteine ([NAC] 20 mM, Sigma) to scavenge free radicals. After 1 hour, cells were infected with GAS (MOI = 4), and plates were spun down to promote bacterial-macrophage interaction. After 20 minutes, gentamicin was added to a final 100-µg/mL concentration to kill extracellular bacteria. At indicated time points, culture media was aspirated, and cells were washed twice in PBS. Cells were then lysed in 0.05% Triton X-100, diluted in PBS, and plated using the drip method onto THY agar plates. Intracellular CFU was enumerated after overnight incubation at 37°C.

### Nitroblue tetrazolium (NBT) reduction

Reduction of NBT, coupled with phenazine methosulfate (PMS) and NADH, with the modifications of Brissac et al. (19). Reactions were performed in 96-well plates in a final volume of 200 µL per well. A mix of nicotinamide adenine dinucleotide reduced (NADH, 66 µM; Sigma), NBT (43 µM; Sigma), and HA (0, 0.1, 1, 2 mg/mL; Sigma) was freshly prepared in phosphate buffer (40 mM, pH 7.6) and incubated for 2 minutes at room temperature. NBT reduction was started by the addition of 2.7-µM PMS. The plates were read in a VICTOR Nivo (PerkinElmer) plate reader at 37°C. The optical density was monitored at 560 nM every 30 s for 30 minutes, with orbital shaking between readings. HA’s antioxidant capabilities were determined to protect NBT from reduction by PMS compared to the controls with no capsule.

### Measurement of intracellular ROS

Intracellular ROS was measured by reduction of 2′,7′-dichlorofluorescein diacetate (DCFDA; Abcam). THP-1 monocytes were differentiated in a black 96-well tissue culture plate using 200-nM phorbol 12-myristate 13-acetate (PMA) for 48 hours before the experiment (9). On the day of the experiment, cells were stained with DCFDA for 45 minutes according to the manufacturer’s protocol and then stimulated with either 1-µM hydrogen peroxide (H_2_O_2_) or 500 ng/mL of recombinant IL-6 protein (Invitrogen). Measurements were taken using a PerkinElmer VICTOR Nivo multimode microplate reader, measuring absorbance at ex/em 485/535 nM every 10 min at 37°C for 180 min total. ROS induction was measured by calculating the V_max_ of each condition in relative units (RU) per minute.

### Fluorescent microscopy

2 × 10^4^ THP-1 monocytes were seeded into each well of an 8-well Millicell EZ SLIDE (EMD Millipore) and terminally differentiated as stated previously. Cell treatments included recombinant IL-6 (Invitrogen; 500 ng/mL) and H_2_O_2_ (200-µM tert-butyl hydrogen peroxide, Abcam). Following a 30 min pre-treatment with IL-6 or TBHP, cells were stained with CellROX deep red (to visualize oxidative burst, 5 µM; Invitrogen) and anti-tubulin conjugated to Alexa Fluor 488 (Novus biologicals, clone YOL1/34, 1:200) for 30 minutes. Cells were then fixed for 20 minutes in BD Cytofix, washed in PBS three times, and then mounted using SlowFade Diamond Antifade Mountant (Thermo Fisher). Samples were visualized on a Zeiss Axio Observer Z1 microscope using 488-nM and 647-nM filters. Images were processed using ImageJ to merge channels and adjust the intensity, with consistent settings between all samples.

### Neutrophil-killing assays

Whole human blood was collected from healthy adult donors with informed consent and approval from the Institutional Review Board at Emory University. Blood was collected into heparinized Vacutainer tubes, and primary neutrophils were isolated using PolymorphPrep (Axis-Shield). Neutrophil-killing assays were performed as described previously with minor modifications (6). Neutrophils were diluted to 10^5^ cells/mL in RPMI containing 10% FBS with no antibiotic, and 1 mL was seeded into each well of a 24-well plate. Neutrophils were either pre-treated with NAC(20 mM, Sigma) for 1 hour or left untreated. 1 × 10^6^ CFU of GAS were added to each well, and samples were taken 90 minutes postinfection for dilution plating. CFU was enumerated after overnight incubation of THY agar plates at 37°C.

### p22 complex formation

In total, 5 × 10^6^ THP-1 monocytes were treated with 500 pg/mL IL-6 (R&D Systems) in RPMI supplemented with 2% FBS for the indicated times. Whole-cell lysates were prepared by adding NP-40 lysis buffer (Alfa Aesar) supplemented with protease inhibitors [1× Halt Protease Inhibitor Cocktail, EDTA-free (Thermo Fisher), 1-µg/mL pepstatin (Millipore Sigma), and 1-mM PMSF (Millipore Sigma)] and mixed with cells at 4°C for 30 minutes. Lysates were spun at 12,000 × *g* to pellet insoluble material, and supernatants were collected. In addition, 10 µg of protein from each sample was run on Any kD Mini-PROTEAN TGX gels (Bio-Rad), transferred to polyvinylidene fluoride (PVDF), and probed with a p22 antibody (Abcam ab75941). Samples were normalized by taking the ratio of the band intensity of the indicated high molecular weight complex to the p22 band intensity. Relative complex formation was calculated by comparing the ratios of the sample to the uninfected cells.

### *In vivo* infection models

All animal experiments were performed with approval from Emory University’s Institutional Animal Care and Use Committee. Moreover, 6- to 8-week-old wild-type C57BL/6 mice were purchased from Jackson Laboratories; CGD [gp91^phox-/-^ (26), also known as NOX2^-/-^] breeder mice were purchased from Jackson Laboratories, and breeding was maintained on-site. Mice were housed under pathogen-free conditions. The day prior to the infection, fur was removed from the back using an electric razor and hair removal cream. For infections, mice were wiped clean with an alcohol pad and injected intradermally with 10^8^ CFU of GAS on the lower back. After 48 hours, mice were euthanized, and skin lesions and spleens were removed. CFU from lesions was enumerated after homogenizing the tissue in 1 mL of sterile PBS. The lesion surface area was quantified using ImageJ.

### Histology

Skin lesions were fixed in 4% paraformaldehyde, paraffinized, and sectioned at a width of 5 µm. Slide preparation and myeloperoxidase (MPO) staining were performed by the Cancer Tissue and Pathology Shared Resource of Winship Cancer Institute of Emory University and imaged using an RXM automated immunohistochemistry staining platform (Leica Biosystems). Slides were heated for 30 minutes at 60°C, deparaffinized with Bond Dewax Solution, and rinsed with Leica wash buffer. Following deparaffination, the slides were heated to 100°C, incubated for 20 minutes with Leica ER2 (high-pH) antigen retrieval buffer, and then rinsed with Leica wash buffer. Peroxidase block was applied at room temperature for 5 minutes, and the sections were washed with three rinses of wash buffer. The anti-myeloperoxidase (Abcam, 1:1,000) was applied and incubated for 30 minutes at room temperature followed by three rinses of wash buffer. Leica Bond anti-Rb HRP secondary was applied and incubated for 8 minutes, and the detection was completed in combination with the Leica refine 3,3ʹ-diaminobenzidine (DAB) kit, as per manufacturer recommendations. Slides were then dehydrated, cover-slipped, and evaluated by light microscopy with scanned images of the slides. The slides were scanned on a Hamamatsu NanoZoomer HT 2.0 at 40 ×. MPO staining was quantified by using the color deconvolution plugin for ImageJ (27) to measure the ratio of integrated density of MPO+ (brown) to total tissue (hematoxylin-stained; blue).

### Cytokine arrays

Cytokine levels in lesion tissue were measured using the V-PLEX proinflammatory Panel 1 Mouse Kit (Meso Scale Diagnostics). This kit measured expression of the cytokines IFN-γ, IL-1β, IL-2, IL-4, IL-5, IL-6, IL-10, IL-12p70, KC/GRO, and TNF-α. Ten samples from each experimental group were randomly selected for analysis. Homogenized tissue in PBS was centrifuged, and total protein content from cell-free supernatants was quantified using Pierce Coomassie Plus (Bradford) Assay Reagent (Thermo Fisher). For cytokine arrays, the total protein was normalized to 40 µg per sample. Quantification and analysis were performed by the Emory Multiplexed Immunoassay Core (EMIC) using a Meso QuickPlex SQ120.

## RESULTS

### IL-6 induces ROS

**Fig 1:**
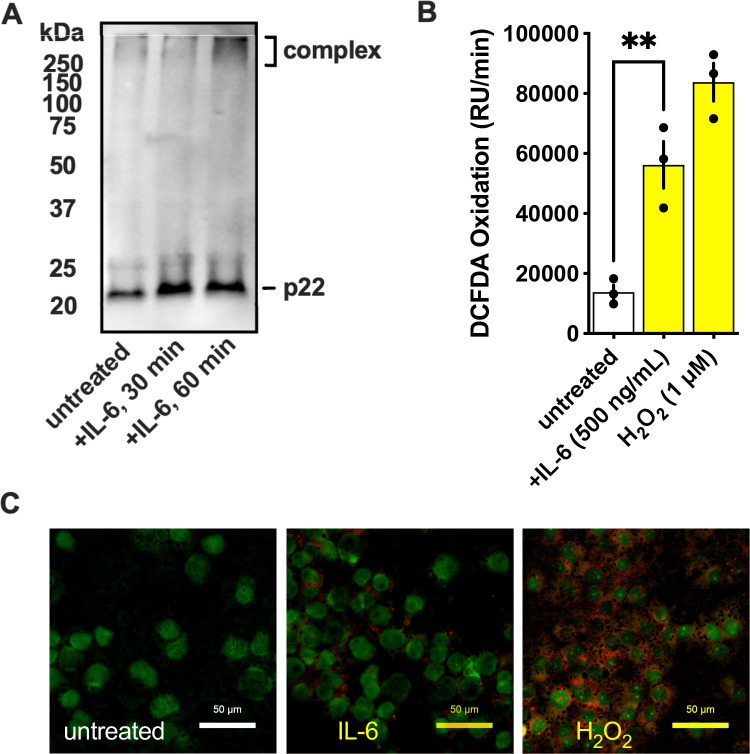
IL-6 induces ROS. (**A**) THP-1 macrophages were treated with IL-6, and cell lysates were examined by Western blot with an anti-p22 antibody. (**B**) Measurement of oxidation by THP-1 human macrophages 30 minutes posttreatment with IL-6, with H_2_O_2_ control. Statistical significance was measured by two-way ANOVA with multiple comparisons. **P < 0.005, (**C**) THP-1 macrophages were treated with IL-6 (500 ng/mL; middle) or H_2_O_2_ (200 µM; right), and relative ROS production was visualized using CellROX deep red (red) in individual cells (anti-tubulin; green) by fluorescent microscopy.

A major mechanism by which activated macrophages kill bacteria is through the production of ROS. Since we previously observed IL-6 enhanced the killing of GAS (9), we investigated whether changes in ROS production contributed to this effect. Phagocytes use a complex formed by NOX2 and p22 to oxidize intracellular NADPH and reduce extracellular oxygen to produce ROS. By Western blotting, we observe the formation of this complex rapidly upon IL-6 treatment (Fig. 1A). Next, we used a dye that fluoresces when oxidized, DCFDA, to measure ROS production by THP-1 human macrophages. The DCFDA oxidation rate was significantly increased by IL-6 stimulation (Fig. 1B), demonstrating that IL-6 can specifically induce ROS. We confirmed this by fluorescent microscopy using CellROX deep red to detect ROS production within THP-1 macrophages. CellROX fluorescence was increased when stimulated with a physiologic IL-6 concentration (500 ng/mL) previously seen to increase macrophage killing of GAS (9) or treated with exogenous H_2_O_2_ (200 µM), relative to untreated cells (Fig. 1C). Together, these data demonstrate that IL-6 can induce ROS, which may participate in the killing of GAS.

### Capsule production correlates with ROS resistance

**Fig 2:**
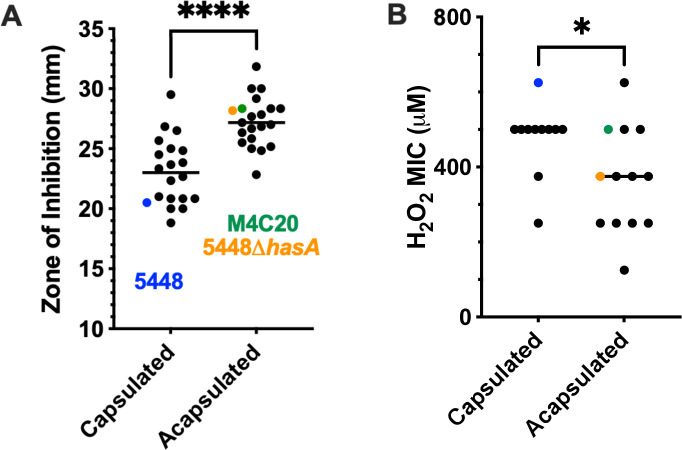
Acapsular strains of GAS are more susceptible to peroxide. (**A**) Hydrogen peroxide shows greater zones of inhibition for acapsular (−) strains of GAS (*n* = 21) than encapsulated (+) strains of GAS (*n* = 20). Each data point represents a single clinical isolate is the average between two experiments each with three technical replicates. (**B**) Capsule-producing GAS strains have an increased minimum inhibitory concentration (MIC) for hydrogen peroxide. The lines are the median values. Statistical significance was determined using an unpaired two-tailed *t*-test. *P < 0.05, ****P < 0.0001.

GAS does not produce catalase (28), and the susceptibility to ROS like hydrogen peroxide varies between strains (29, 30). Since strains of the M4 serotype that are sensitive to IL-6-mediated killing (9) also lack a capsule (10), we tested the hypothesis that a capsule protects against ROS-mediated killing by measuring the susceptibility of a panel of clinical isolates to hydrogen peroxide on both solid and liquid media. The widely studied M1T1 GAS strain 5448 hyperinvasive strain was included as a control (23). Each strain was tested for capsule production by an enzyme-linked binding protein assay against hyaluronan (31, 32). GAS clinical isolates that were naturally acapsular (M4, M22, M28, and M89) had significantly larger zones of inhibition on THY plates spotted with hydrogen peroxide than encapsulated GAS isolates (of M1, M3, M6, M11, M12, and M88 serotypes), indicating enhanced susceptibility (Fig. 2A). To examine whether capsule loss would increase susceptibility, we tested a mutant of the M1T1 reference strain 5448 deficient in the hyaluronate synthase (5448∆*hasA*) (31). This mutant has a significantly increased susceptibility to peroxide, confirming a contributing role for capsule specifically in the variable sensitivity between clinical isolates (Fig. 2A). We also employed a modified MIC assay using alamarBlue, a colorimetric reagent that measures cellular respiration, to determine each strain’s susceptibility to hydrogen peroxide (24). The MIC of strain 5448∆*hasA* was approximately half of the MIC of wild-type 5448 (Fig. 2B). Consistent with the plate assay, acapsular clinical isolates trended to lower MICs toward peroxide (median 375 µM) compared to 5448 and other encapsulated clinical isolates of GAS (median 500 µM). Together, these data support the hypothesis that the hyaluronic acid capsule produced by some GAS strains provides a mechanism to evade killing by ROS *in vitro*.

### Hyaluronic acid detoxifies ROS

**Fig 3:**
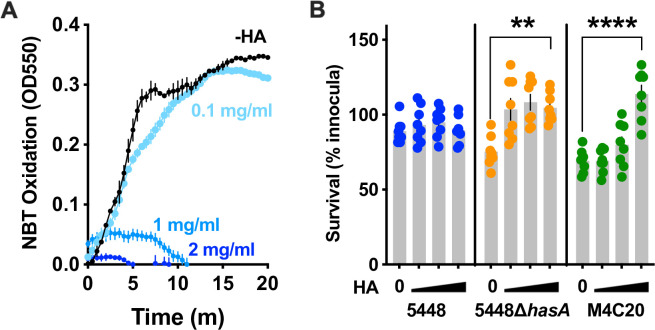
Hyaluronic acid directly protects against peroxide-mediated killing. (**A**). Measurement of NBT reduction in the presence of hyaluronic acid titrations. Data background from the no-PMS-negative control was subtracted. (**B**) Killing of GAS by 10-mM hydrogen peroxide in the presence of titrations of exogenous hyaluronic acid (0, 0.1, 1, 2 mg/mL). Data are expressed as the mean ± SEM. Statistical analysis was done by one-way ANOVA with Tukey’s multiple comparisons posttest. **P < 0.005; ***P < 0.0005; ****P < 0.0001.

Extracellular polysaccharides of many bacterial species can act as antioxidants (33). Human hyaluronic acid is a known antioxidant (34), so we hypothesized that the capsule of GAS, made of hyaluronic acid, would similarly have this activity. Specifically, this would posit that exogenous hyaluronic acid could sacrificially protect bacteria from oxidation and that this would not strictly require forming a barrier around the cell. First, we measured the reduction of NBT paired with PMS and NADH as previously described (25). To this, titrations of hyaluronic acid were added, and the decreases in NBT reduction were measured (Fig. 3A). In addition, 1-mg/mL hyaluronic acid was sufficient to prevent the NBT reduction, demonstrating the ability of hyaluronic acid to directly act as an antioxidant. To expand on these observations, we next examined the ability of exogenous hyaluronic acid to rescue the growth of GAS in the presence of hydrogen peroxide. Moreover, 5 × 10^7^ CFU of GAS were incubated with 10-mM hydrogen peroxide and titrations of hyaluronic acid, and 1 mg/mL was sufficient to restore the growth of the acapsular strains 5448∆*hasA* and M4C20 (Fig. 3B). The growth of encapsulated strain 5448 was unaffected by exogenous hyaluronic acid, suggesting it synthesizes enough to be sufficiently protective. These observations are consistent with our hypothesis that the capsule of GAS can protect against ROS.

### ROS kills GAS *in vitro*

**Fig 4:**
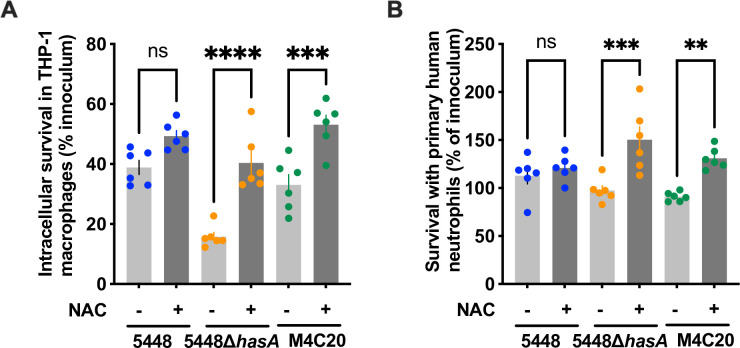
(**A**) Intracellular survival of GAS within THP-1 macrophages with the addition of 20-mM NAC, a ROS scavenger. (**B**) Survival of GAS incubated with human primary neutrophils with the addition of 20-mM NAC, a ROS scavenger. Statistical significance was measured using ordinary one-way ANOVA. **P < 0.005; ****P < 0.0001; ns, no significance. Data are expressed as the mean ± SEM.

To determine whether ROS kills acapsular GAS during interactions with immune cells, we modeled infections *in vitro* with both macrophages and neutrophils. These cells are recruited during early GAS infection and are major producers of ROS (1). Intracellular survival within macrophages is important for GAS pathogenesis and is a location where they will potentially be exposed to bactericidal ROS (9, 35, 36). NAC is used experimentally and clinically as a ROS scavenger and could ameliorate the requirement for a capsule if its primary function is to detoxify ROS. THP-1 monocytes were terminally differentiated into macrophages and treated with either 20-mM NAC or left untreated and then infected with 8 × 10^4^ CFU (MOI = 4) of GAS strains M1T1 5448, 5448∆*hasA*, or M4C20. After 20 minutes, gentamicin was added to a final 100-µg/mL concentration to kill extracellular bacteria, then intracellular bacteria were harvested at 90 minutes, and survival was determined by CFU plating. Intracellular growth of acapsular GAS (5448∆*hasA* or M4C20) was significantly increased in macrophages treated with NAC when compared to untreated macrophages (Fig. 4A). Similarly, primary neutrophils from human donors were treated with NAC and infected at MOI = 10. As with macrophages, acapsular strains of GAS experience significantly increased survival with NAC treatment (Fig. 4B). Encapsulated M1T1 5448 GAS showed no significant difference in growth within either cell type during NAC treatments, demonstrating that depletion of ROS is sufficient to allow growth of acapsular GAS and that capsule is sufficient to protect against ROS-mediated intracellular killing.

### Phagocyte ROS is not essential for killing GAS in skin and soft tissue infection

**Fig 5:**
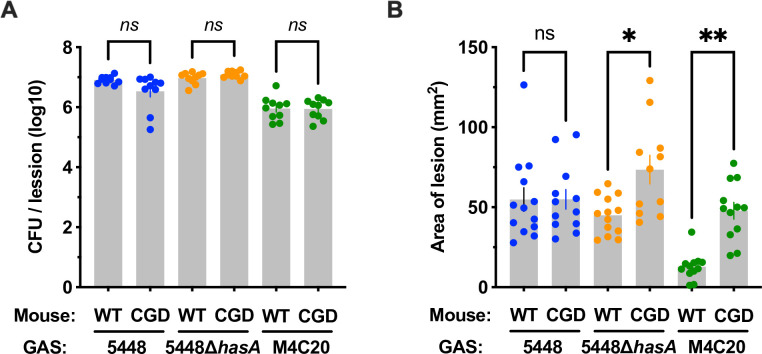
The role of capsule during invasive skin infection of chronic granulomatous disease mice. Wild-type C57BL/6 or gp91^phox-/-^ (CGD) mice were injected intradermally with 10^8^ CFU of GAS 5448, 5448∆*hasA*, or M4C20, and infection was monitored for 48 hours. (**A**) CFU was enumerated from homogenized whole lesions by dilution plating. (**B**) Lesion size was measured. Each data point represents a single mouse, and the results are pooled across three independent experiments. Data are expressed as mean ± SEM. Statistical significance was determined by an unpaired two-tailed *t*-test. *P < 0.05; ns, no significance.

Based on this *in vitro* evidence that the GAS capsule is an antioxidant for ROS produced by immune cells, we next examined survival in an *in vivo* infection model using gp91^phox-/-^ [chronic granulomatous disease model (CGD)] mice that are deficient in phagocyte ROS production and neutrophil function (26). Using a subcutaneous skin infection model, Lei et al. previously observed similar replication of wildtype M1T1 (encapsulated) GAS in wild-type and gp91^phox-/-^ mice (37). We hypothesized that this could be due to the capsule preventing phagocytosis and protecting from ROS and that acapsular bacteria (5448∆*hasA* and M4C20) would have an attenuation in wild-type mice that would be reversed in gp91^phox-/-^ mice. Mice were inoculated intradermally with 1 × 10^8^ CFU of wild-type 5448, 5448∆*hasA*, or M4C20, and skin lesions were harvested and processed for CFU enumeration after 48 hours. In contrast to these expectations, the bacterial load within lesions was nearly identical between wild-type and gp91^phox-/-^ mice regardless of whether they were infected with either encapsulated or acapsular bacteria (Fig. 5A).

In contrast to the total bacterial number, the size of the skin lesions formed in gp91^phox-/-^ mice was significantly greater (Fig. 5B). Lesions in gp91^phox-/-^ mice by 5448∆*hasA* averaged nearly twice as large, and for M4C20, four times larger, than in wild-type mice. Infection of gp91^phox-/-^ mice typically involved a greater region of tissue, with more dispersed bacteria or pathological inflammation that contributed to forming the skin lesion. Despite this difference in apparent pathology, the CFU for each strain did not vary between mouse genotypes (Fig. 5A). Therefore, these findings suggest that phagocyte ROS is not essential for the direct killing of GAS at this site of infection but still had a contribution to the immune response. Furthermore, these indicate that the capsule was not an essential virulence factor in this model, potentially in part due to the lack of killing by phagocyte ROS that was observed.

### Proinflammatory cytokines do not account for bacterial survival or lesion differences

**Fig 6:**
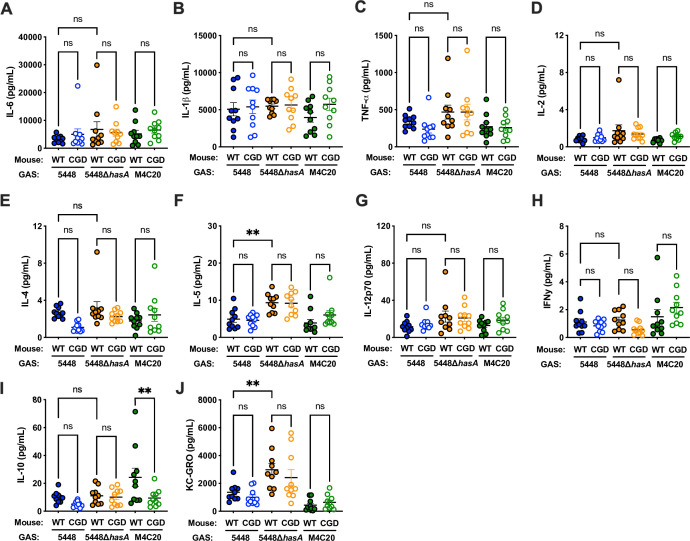
Wild-type and gp91^phox-/-^ mice produce similar pro-inflammatory cytokine profiles. (**A–J**) Cytokines from cell-free lysates from infections of wild-type C57BL/6 or gp91^phox-/-^ mice with 10^8^ CFU of GAS 5448, 5448∆*hasA*, or M4C20 (from Fig. 5) were measured 48 hours postinfection by V-PLEX proinflammatory cytokine panel 1 (MSD). Each point represents a lesion from a single mouse. Data are expressed as the mean ± SEM. Statistical analysis was performed using ANOVA with Tukey posttest. ** =*P* < 0.005

Since bacterial numbers remained constant, we hypothesized that the change in lesion size in CGD mice could result from misregulated inflammation during infection. To investigate this hypothesis, we used a multiplexed cytokine array to measure the levels of pro-inflammatory cytokine expression at the infection site. IL-6, an inducer of ROS that we showed is important in promoting the killing of acapsular GAS (9), was produced at similar levels between all infections (Fig. 6A). This excluded the possibility that deficiency in IL-6 signaling was a contributing factor to smaller lesion sizes produced by acapsular GAS. IL-1β and TNF-α, two additional acute-phase proinflammatory cytokines important in killing GAS in this model (4) were also similarly induced across conditions (Fig. 6B and C). Of the cytokines examined (Fig. 6A through J), only the anti-inflammatory cytokine IL-10 was significantly different between mouse genotypes; it was elevated in wild-type mice infected with M4C20 over the gp91^phox-/-^ (CGD) mice (Fig. 6J). While this increased IL-10 correlates with decreased lesion size in M4C20-infected CGD mice (Fig. 5B), this does not explain the capsule- or ROS-dependent effects. At an equivalent bacterial burden, the capsule mutant of 5448 (∆*hasA*) trended to induce greater inflammation; for the eosinophil activator IL-5 (Fig. 5F) and the neutrophil chemokine KC-GRO (CXCL1; Fig. 5J), this reached statistical significance (*P* = 0.0049, 0.0063 respectively). This could reflect a contribution of the capsule to dampening proinflammatory signaling, such as by shielding pathogen-associated molecular patterns on the GAS surface or hyaluronic acid engaging inhibitory cell receptors. Other cytokines measured, but not significantly different across groups, included IL-2 (Fig. 5D), IL-4 (Fig. 5E), and IL-12p70 (Fig. 5G), all cytokines involved in lymphocyte function, and IFN-γ (Fig. 5H), an important activator of macrophages and other cells. Thus, the magnitude or repertoire of inflammatory response does not account for differences in CFU or lesion formation.

### Role of neutrophils in the immune response to capsule-deficient GAS

**Fig 7:**
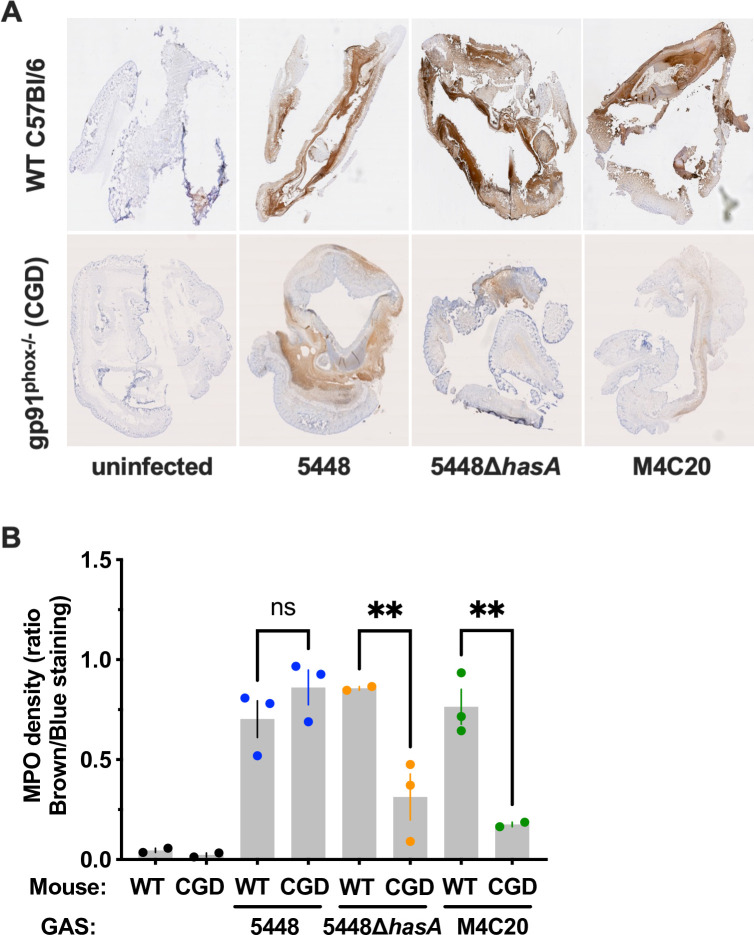
Skin lesions from gp91^phox-/-^ mice infected with acapsular GAS have decreased levels of myeloperoxidase compared to wild-type mice. MPO production was determined using immunohistochemistry with antibodies probing for MPO (Abcam). (**A**) Representative images of lesions stained with anti-MPO (*n* = 3 for each group). (**B**) MPO levels were quantified using color deconvolution in ImageJ. Values represent the ratio of the intensity of DAB-stained cells to the intensity of total cells and are plotted as the mean ± SEM. Each data point represents a single lesion. Statistical significance was determined using ANOVA with Tukey posttest. ns, no significance, **P < 0.01.

Neutrophils are significant mediators of the immune response to GAS, pathological inflammation, and wound healing (16, 38, 39). MPO is a standard neutrophil marker whose expression is not impacted by gp91^phox^ deficiency. Mice were infected as in Fig. 5 and 6, and tissue was fixed, sectioned, stained with an anti-MPO antibody and DAB, and then counter-stained with hematoxylin to visualize and quantify MPO production within tissues (Fig. 7A). The ratio of MPO-positive (brown) tissue to total (hematoxylin-stained, blue) was used to normalize between tissue sections for evaluation of neutrophil numbers. Lesions from wild-type mice all had similar ratios between both encapsulated and acapsular strains (Fig. 7B). However, in CGD mice, neutrophils within lesions had significantly lower MPO + cell ratios when infected with acapsular GAS (5448∆*hasA* or M4C20). Wild-type or gp91^phox-/-^ mice infected with GAS strain 5448 did not have any statistically significant differences in MPO + ratios (Fig. 7B). These data support the hypothesis that capsule production by GAS, as well as ROS production within the host, contribute to wound healing during GAS infection, and neutrophils may largely modulate this in an MPO-dependent manner.

## DISCUSSION

In this study, we aimed to determine the molecular mechanisms by which IL-6 promotes the killing of acapsular GAS. We show that IL-6 induces greater production of ROS (Fig. 1) and that the hyaluronic acid capsule protects against this killing (Fig. 2). ROS-mediated killing of GAS is of great interest since GAS does not produce catalase for the decomposition of hydrogen peroxide as many other bacteria do. Nonetheless, it is quite resistant to peroxide, even producing it as a metabolic byproduct, suggesting it has other mechanisms contributing to its resistance (29). These may include the alkyl hydroperoxide reductase *ahpC*, glutathione peroxidase *gpoA*, and NADH oxidase *noxA*, which are induced by oxidative stress and contribute to virulence through these or other activities (28, 40, 41). Notably, *ΔahpC* GAS have no growth attenuation but form a significantly smaller lesion in a skin infection model (42), parallel to our observations on capsule-deficient GAS (Fig. 5). This could suggest that a mechanism for ROS-sensitive GAS to persist *in vivo* is to remain more spatially confined, or alternatively, that ROS is more effective against bacteria disseminating from the infection locus.

These differences may partly be due to the biology of neutrophils, the major ROS-producing immune cells. The importance of neutrophils during GAS infection is well-established in both *in vitro* and *in vivo* infection models (6, 16, 29, 37, 38, 43, 44). Since the production of ROS is such a major function of neutrophils, it would be expected that gp91^phox-/-^ mice would be significantly impaired in their ability to kill GAS. However, our findings (Fig. 5) agree with prior work (37) to suggest that this is not their primary GAS-killing mechanism during infection of the skin. Lei et al. go on to show that gp91^phox-/-^ mice are more sensitive to pulmonary infection by GAS (37). While GAS pulmonary infections are rare in humans, this provides proof of principle that at other body sites, neutrophil ROS may be more effective against GAS and that this is potentially why these infections are uncommon. Other neutrophil antimicrobials such as reactive nitrogen species, cathelicidin, elastase, and neutrophil extracellular traps may have a larger role in the control of skin and soft tissue infections than ROS. Nonetheless, ROS are also important signaling molecules that control redox-sensitive processes including transcription of NF-kB, cell migration, phagocytosis, cell death, and formation of neutrophil extracellular traps (45). Thus, ROS have the potential to contribute to the immune response outside of their direct antimicrobial function.

Despite the multitude of benefits that come from capsule production, it is unclear how strains of GAS that do not produce it persist in the human population and remain capable of causing severe invasive disease. In addition to protection from ROS, the capsule can act as a shield against complement, antimicrobial peptides, and opsonization and engages the host hyaluronic acid receptors CD44 and LYVE1 to promote colonization and invasion of non-phagocytic cells (28, 46 – 50). Supporting these essential roles, capsule mutants of M3 (51), M18 (52), and M24 (53) GAS are attenuated in mouse skin and soft tissue models of infection. Hurst et al. recently went on to show that the attenuation of M18 capsule mutants of M18 GAS can be reversed by neutrophil ablation, suggesting another essential function for the capsule is to protect it from neutrophil-mediated killing (16). Yet, all M4 and M22 GAS remain virulent despite naturally lacking the operon required for capsule biosynthesis and are in fact increasingly associated with severe infections in the USA (10, 32, 54). Notably, loss of capsule has been associated with increased expression of streptolysin O and the NAD-glycohydrolase, associated with hypervirulence (55, 56). Henningham et al. see that the introduction of the *has* operon to acapsular M4 GAS is sufficient for capsule production and increased neutrophil resistance *in vitro*, yet does not increase virulence *in vivo* (32). Furthermore, attenuation of capsule mutants is not seen in all models. Our results (Fig. 5) and those of Cole et al. (57) find that a capsule is not required in models of invasive infection using M1 GAS, the serotype most commonly associated with severe infections (58). Together, these data argue that GAS may have redundant mechanisms for dealing with immune effectors, suggesting that the need for a capsule could be abrogated in some strains through yet unknown compensatory virulence factors present in hypervirulent M1 and encapsulated strains.

We further show that the capsule may not only be a barrier but can act as a sacrificial antioxidant to detoxify ROS directly. In 1979, Cleary and Larkin provided evidence supporting this, showing that the capsule slows the uptake of O_2_ and that GAS can produce millimolar levels of peroxide (30). Additionally, they show an accumulation of 60× more peroxide in cultures treated with hyaluronidase, consistent with the bacterial hyaluronan acting as a redox sink for microbial-produced ROS. Using isogenic mutants and a range of clinical isolates, we show that genetically acapsular GAS are significantly more susceptible to peroxide *in vitro* (Fig. 2). The addition of ROS scavengers was sufficient to rescue the growth of acapsular GAS from killing by phagocytes (Fig. 4). Together, these argue for a role in capsule in ROS detoxification. This is also in line with the observation of antioxidants that carbohydrates of many species (33), including the capsules of *Cryptococcus neoformans* (17), *Salmonella* Typhimurium (18), and *Streptococcus pneumoniae* (19), can be antioxidants. Since the GAS capsule is structurally identical to the hyaluronic acid in human connective tissue, this is considered important in molecular mimicry that shields the bacterium from immune detection (59). Subsequently, the capsule oxidation products may mimic those seen in human tissue during inflammatory diseases such as rheumatoid arthritis (26) and have similar pathological or inflammatory effects.
